# Extremely Weak Electro-Optic Kerr Effect in Methyl Silicone Oils

**DOI:** 10.3390/ma17081850

**Published:** 2024-04-17

**Authors:** Marek Izdebski, Rafał Ledzion, Szymon Węgrzynowski

**Affiliations:** Institute of Physics, Lodz University of Technology, Wólczańska 217/221, 93-005 Lodz, Poland; rafal.ledzion@p.lodz.pl (R.L.); szymon.wegrzynowski@edu.p.lodz.pl (S.W.)

**Keywords:** methyl silicone oils, electro-optic Kerr effect, polarimetric method, immersion liquid

## Abstract

The electro-optical properties of methyl silicone oils with viscosities ranging from 10 to 10,000 cSt have been studied extensively to verify their suitability as immersion liquids. Immersion liquids are often used in nonlinear optics to protect hygroscopic crystals from moisture, reduce multiple reflections, and protect against electrical breakdown. However, the lack of experimental data makes it difficult to select an optimal liquid that does not exhibit a significant electro-optical Kerr effect in the fringing electric field around the electrodes on the crystal. Electro-optical measurements were performed using an improved dynamic polarimetric method, which compensates for the measurement errors caused by inaccurate positioning of the electro-optical modulator’s operating point on its transmission characteristics. The values of the Kerr coefficient ranged from −8.83 × 10^−16^ to −6.79 × 10^−16^ m V^−2^ for all oil samples, at temperatures from 25 to 80 °C and frequencies from 67 to 1017 Hz. These exceptionally low values, together with a low dielectric constant, very good transparency, and high chemical stability, make methyl silicone oils highly suitable as immersion liquids. The Kerr coefficient and other electro-optical coefficients increased with increasing temperature. This unusual result cannot be adequately explained by Buckingham’s molecular theory of the Kerr effect.

## 1. Introduction

The continuing improvement of techniques for measuring electro-optic effects requires sources of measurement errors to be considered that were previously neglected. One such source of measurement errors is the contribution of the immersion liquid to the electro-optical measurements of solid samples. An immersion liquid is often necessary to protect hygroscopic crystals against moisture. For example, immersion liquids are used to protect KDP and KD*P crystals, which are very commonly used in nonlinear optics. The immersion liquid also suppresses the effect of multiple reflections on the faces of the solid sample, reduces light scattering on surface irregularities, and protects against electrical breakdown. However, the lack of experimental data makes it difficult to select an optimal liquid that does not exhibit a significant electro-optical Kerr effect in the fringing electric field around the electrodes on the crystal.

The aim of this work is to provide reliable measurements of the quadratic electro-optic effect as a function of temperature and frequency of the applied electric field for several methyl silicone oils with viscosities ranging from 10 to 10,000 cSt. In order to perform measurements for many combinations of temperature, modulating field frequency, and oil viscosity, we have developed a new approach to electro-optical measurements that combines measurement speed with good accuracy. We propose the use of a standard polarimetric setup along with a new measurement procedure, in which the measurements are carried out for two operating points on the transmission characteristics of the electro-optic modulator. As will be demonstrated here, it is necessary to control only the relative position of these two points, for example, using an analyzer mounted on a stepper motor, which is much more precise and easier than controlling the absolute position of a single operating point, as in the traditional approach.

Our results show that the Kerr coefficient ranges from −8.83 × 10^−16^ to −6.79 × 10^−16^ m V^−2^ for all oil samples in the entire temperature range from 25 to 80 °C and frequencies from 67 up to 1017 Hz. Since these values are very small and are smaller than almost all previously reported ones for many other liquids (e.g., [1,2,3,4,5,6,7,8]), methyl silicone oils seem to be particularly suitable as immersion liquids. There are many other transparent liquids with low Kerr coefficients of the order 10^−15^ m V^−2^, but most are unsuitable for use as immersion liquids because of their volatility, flammability, toxicity, low boiling point, hydrophilicity, or electrical conductivity. Organic oils may also have unstable electro-optical properties, which are caused by aging [8] or by orientational ordering of molecules in an electric field, which can depend not only on the current temperature but also on the thermal history of the oil [9]. However, methyl silicone oils do not appear to have these problems. Moreover, the low dielectric constant of these oils allows for an almost uniform electric field inside the solid sample between the electrodes.

We found that the absolute values of all types of electro-optic coefficients for all oil samples increase with increasing temperature, which cannot easily be explained by current theories. For liquids, the classical statistical-mechanical theory of Buckingham and Raab [1] predicts a decreasing temperature dependence of the molecular Kerr constant, which can be expressed as

(1) $$K_{m}=A_{0}+A_{1}T^{-1}+A_{2}T^{-2},$$

where $A_{0}$, $A_{1}$, and $A_{2}$ are constants specific to a substance. The constant *A*_0_ is proportional to the second-order hyperpolarizability of the molecule, *A*_1_ is related to the first-order hyperpolarizability and polarizabilities for the frequency of the incident light and for the static electric field, and *A*_2_ is related to the permanent dipole moment. Dependence (1) has been experimentally confirmed for nitrobenzene [2], as well as for several organic liquids belonging to the homologous series of ketone, aldehyde, nitriles [3], and many others. In the case of molecules without permanent dipole moments, the term $A_{2}T^{-2}$ vanishes. A linearly decreasing dependence of $K_{m}$ on $T^{-1}$ has been confirmed, for example, for carbon disulfide, mesitylene [1], benzene, toluene [4], and perfluoropolyether Fomblin M03 [5]. Several other decreasing dependencies of the Kerr *K* coefficient have been proposed, based on experimental data for specific liquids, such as $K=\sum_{k=0}^{3}A_{k}/T^{k}$ for water [6], the Van ’t Hoff-type expression $\log K=A_{0}+A_{1}T^{-1}$ for binary liquid mixtures of aprotic-aprotic molecules [7], or the linear dependence $K=A_{0}+A_{1}T$, with $A_{1}<0$, for fresh and aged transformer oil [8]. It should be noted that the mentioned papers provided only absolute values of $K_{m}$ or *K* without determining their sign. To our knowledge, very little data on methyl silicone oils have been available to date. The increasing temperature dependence of the absolute value of the Kerr coefficient was previously reported for a single sample of Polsil OM-3000 oil [10], but the obtained values are several times overestimated compared to the results obtained later in ref. [11] and in this study. Work [11] focuses on the fact that the Kerr coefficient at room temperature has slightly smaller values for oils of lower molecular mass, and no temperature or frequency dependences are measured within the study. In the case of solid crystals, the typical temperature dependencies of various electro-optical coefficients also decrease when the measurements do not involve phase transitions [12,13,14,15,16].

The decreasing temperature dependence of the dielectric constant in methyl silicone oils means that electro-optic coefficients traditionally considered to be less temperature-dependent, such as those defined in terms of polarization by Geusic [12] and Miller [13] or the molecular Kerr constant [1], appear to grow even faster with increasing temperature than the usual electro-optic coefficients. Our results further show that the frequency dependence of the Kerr coefficient in methyl silicone oils is relatively weak.

## 2. Materials and Methods

**Figure 1:**
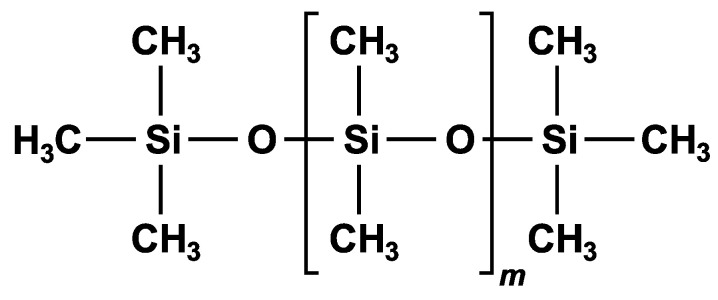
Chemical structure of methyl silicone oil (polydimethylsiloxane/PDMS).

Methyl silicone oils are liquid organosilicon polymers composed of alternating silicon and oxygen atoms, which form a linear molecule with two methyl groups attached to each silicon atom (Figure 1). These polymers have a wide range of useful properties, including transparency, very good dielectric properties, good surface wetting, hydrophobicity, and resistance to both low and high temperatures as well as to atmospheric and chemical factors. As a result, silicone oils have found numerous applications, including as liquid dielectrics, lubricants, hydraulic oils, heat exchanger oils, and as additives for cosmetics, paints, varnishes, car body creams, shoe and floor polishes, and furniture polishes.

There are many silicone oils with backbone chains consisting of alternating silicon and oxygen atoms. Multiple groups can be attached to tetravalent silicon centers. Some of these groups may be highly active, making them unsuitable for applications requiring resistance to multiple factors. Since the methyl group is highly stable and inactive, this determines the very low activity of the entire polymer molecule containing only methyl groups.

Methyl silicone oils are produced in a wide range of viscosities. The viscosity of the oil is related to the average number of dimethylsiloxane groups in the molecule (Table 1). The measurements presented in this paper were made for six samples of POLSIL^®^ OM-10, OM-50, OM-300, OM-1000, OM-3000, and OM-10000 oils, where the number means the viscosity given in cSt at 25 °C. All tested oils were manufactured by Silikony Polskie Ltd., Nowa Sarzyna, Poland.

To measure the electro-optical properties of the methyl silicone oils, we used a standard polarimetric system consisting of a laser, a linear polarizer, the sample, a retardation plate, and a linear analyzer. In our experiment, an electric field **E** was applied to the sample in a direction perpendicular to the light beam. We assume that the azimuths of all components are given relative to the direction of the field **E**, which indicates the 0° azimuth. In an optimal case, the dependence of the transmitted light intensity *I* on the phase difference induced in the sample by the applied field should be strongest and almost linear. When the sample is non-dichroic and optically inactive, and when the azimuth of the fast wave in the sample subjected to the field is 0° or 90°, the azimuth of the polarizer should be α = −45° or +45°, the azimuth of the analyzer should be β = −45° or +45°, and the azimuth of the fast wave in the retardation plate should be θ = 0° or 90° [18]. These eight optimal measurement configurations lead to the following dependency for a theoretical system composed of ideal elements:

(2) $$I=\frac{1}{2}I_{max}\left[1\pm \cos\left(\gamma -q\Gamma \right)\right],$$

where $I_{max}$ is the maximum value of *I*; the signs “+” and “−” correspond to the parallel (α=β) and perpendicular (α=−β) polarizers, respectively; γ and Γ are the phase differences appearing in the retardation plate and in the sample, respectively; and q=+1 for θ = 0° or q=−1 for θ = 90°. Here, Γ is used to represent the phase difference between a 0° polarized wave and a 90° polarized wave, which can take both positive and negative values, while γ always represents a positive value. It is convenient to consider Γ as the sum of the field-free $\Gamma_{0}$ value and the small change ∆Γ induced in the sample by the field. When the symmetry of the sample excludes the linear electro-optic effect, we obtain

(3) $$\Gamma =\Gamma_{0}+∆\Gamma =\Gamma_{0}+2\pi LKE^{2},$$

where *L* is the length of the sample and *K* is the Kerr coefficient.

Let us now consider a dynamic method with a sinusoidal modulating field, as follows:

(4) $$E(t)=E_{0}\sin\omega t,$$

where the amplitude of the applied field results from the RMS voltage $U_{m}$ applied to the electrodes with a distance *d*, as follows:

(5) $$E_{0}=\sqrt{2}U_{m}/d.$$

The changes in the phase difference caused by the quadratic electro-optic effect are usually very small. Therefore, $\sin\left(∆\Gamma \right)\approx ∆\Gamma$, $\cos\left(∆\Gamma \right)\approx 1$, and the contribution of the rectified quadratic electro-optic effect $\pi LKE_{0}^{2}$ is negligibly small compared with the total field-free phase difference $\gamma -q\Gamma_{0}$. These approximations and Equations (2)–(4) lead to

(6) $$\frac{I}{I_{max}}=\frac{1}{2}\pm \frac{1}{2}\left[\cos\left(\gamma -q\Gamma_{0}\right)-q\pi LKE_{0}^{2}\sin\left(\gamma -q\Gamma_{0}\right)\cos\left(2\omega t\right)\right].$$

The light passing through the modulator causes a voltage U~I at the photodetector output. Equation (6) shows that the voltage *U* contains a DC component $U_{0}$ and a second harmonic component of the modulating field. Following the convention used in lock-in amplifiers, we describe the second harmonic with the non-negative RMS voltage $U_{2\omega }$ and the phase $\varphi_{2\omega }\in (-180{}^{\circ};+180{}^{\circ}]$ as

(7) $$U=U_{0}+\sqrt{2}U_{2\omega }\sin\left(2\omega t+\varphi_{2\omega }\right).$$

We easily find from Equations (6) and (7) that $\varphi_{2\omega }$ takes only two values, −90° and +90°, and its sign is related to the configuration of the experimental setup and the sign of the Kerr coefficient. To simplify the notation, we define the modulation depth at the second harmonic frequency as a signed value, as follows:

(8) $$m_{2\omega }=sgn\left(\varphi_{2\omega }\right)\frac{U_{2\omega }}{U_{0}}\;.$$

From Equations (5)–(8), we find that

(9) $$K=-\frac{\pm qd^{2}m_{2\omega }}{\sqrt{2}\pi LU_{m}^{2}}\;\frac{1\pm \cos\left(\gamma -q\Gamma_{0}\right)}{\sin\left(\gamma -q\Gamma_{0}\right)}\;.$$

In practice, measurements often involve samples that are not expected to be naturally birefringent, such as isotropic liquids and solids or uniaxial crystals with light propagating along the optical axis. In such cases, $\Gamma_{0}=0$, and a quarter-wave plate is traditionally used to ensure optimal measurement conditions at γ=90°. If this condition is satisfied exactly, Equation (9) is simplified to

(10) $$K^{\prime }=-\frac{\pm qd^{2}m_{2\omega }}{\sqrt{2}\pi LU_{m}^{2}}\;.$$

According to Equation (10), all eight optimal measurement configurations should lead to the same result for $K^{\prime }$. However, in our measurements, we often observed significant systematic discrepancies between the results obtained for different configurations, which cannot be explained by errors in the measuring instruments or by an imperfect alignment of the sample and optical elements. We found that this problem was mainly due to deviations in the total field-free phase difference $\gamma -q\Gamma_{0}$ from the ideal value of 90°, which is difficult to avoid in practice. Consequently, Formula (10) yields an inaccurate result for $K^{\prime }$, instead of the exact *K* (9). The relative error is

(11) $$∆K=\frac{\left|K^{\prime }\right|-\left|K\right|}{\left|K\right|}=\frac{\sin\left(\gamma -q\Gamma_{0}\right)}{1\pm \cos\left(\gamma -q\Gamma_{0}\right)}-1.$$

According to our observations, there are three main reasons for the deviation of the actual phase difference $\gamma -q\Gamma_{0}$ from the intended value of 90°:

1. The inaccuracies of commercially available quarter-wave plates are of the order of several degrees or, according to some researchers, up to 10° [19].
2. The mechanical stress in glass and quartz cuvettes may cause a phase difference of up to 4°. This value varies depending on the temperature and where the light beam passes through on the faces of the cuvettes. We observed that even cuvettes of the same type are unique, but the fast-wave azimuth is typically almost parallel or perpendicular to the long edges of the optical windows. Thus, the phase difference introduced by a cuvette simply adds or subtracts from that introduced by the quarter wave plate and the sample in the cuvette.
3. The orientational ordering of elongated particles in an applied electric field also causes a phase difference. For highly viscous liquids in a field with a frequency of tens of hertz or more, we mainly observed the DC component of this phase difference, rather than the harmonic components. We have previously that this phase difference can reach values on the order of several tens of degrees at a distance of 50 mm shown for mineral transformer oil [9]. Even if this effect in a given liquid is weak, it cannot be presumed due to the limited data in the literature.

The actual phase difference introduced by the quarter-wave plate can be measured and included in the calculations based on Equation (9). However, this approach is not suitable for suppressing the errors caused by the unstable birefringences mentioned in points 2 and 3. The solution to this problem proposed in the literature is to compensate for birefringence in a Sénarmont-type system [18], in a photoelastic dynamic retarder used instead of a static quarter-wave plate [20,21], or in a sample using a DC bias voltage [22]. Such systems should be readjusted after changing any parameters that could affect the total phase difference. Because this is time-consuming and difficult, we propose performing measurements without controlling the actual total phase difference in the present study. The systematic error given by Equation (11) can be eliminated by performing measurements for the two orientations of the analyzer while maintaining the positions of the polarizer and quarter-wave plate. These two measurements allow the determination of two modulation depths, $m_{2\omega }^{-}$ and $m_{2\omega }^{+}$, for β = −45° and +45°, respectively, and for the corresponding two imprecise values $K^{\prime -}$ and $K^{\prime +}$ resulting from Equation (10). Considering, for example, a deviation of the total phase difference $\gamma -q\Gamma_{0}$ up to ±10° from the ideal value of 90°, we obtain a relative measurement error Δ*K* (11) of up to −16%...+19%. It is important that when one of the two inaccurate values $K^{\prime -}$ or $K^{\prime +}$ is underestimated, the other is overestimated, and the accurate value *K* must lie between them. Using the basic trigonometric relations in Equations (9) and (10), it can be shown that the geometric mean of $K^{\prime -}$ and $K^{\prime +}$ is independent of $\gamma -q\Gamma_{0}$ and equal to the exact absolute value |*K*|, as follows:

(12) $$\left|K\right|=\frac{qd^{2}}{\sqrt{2}\pi LU_{m}^{2}}\sqrt{\left|m_{2\omega }^{-}m_{2\omega }^{+}\right|\;\frac{1-\cos\left(\gamma -q\Gamma_{0}\right)}{\sin\left(\gamma -q\Gamma_{0}\right)}\;\frac{1+\cos\left(\gamma -q\Gamma_{0}\right)}{\sin\left(\gamma -q\Gamma_{0}\right)}}=\sqrt{K^{\prime -}\;K^{\prime +}}.$$

The sign of *K* can be taken to be the same as the signs of $K^{\prime -}$ and $K^{\prime +}$.

Our proposed method offers good accuracy and speed, which is important for extensive research involving many combinations of various parameters. However, the method is not suitable for samples with high birefringence, which may result in an incorrect sign of *K*. Additionally, when the total phase difference $\gamma -q\Gamma_{0}$ is close to zero, the accuracy of the measurements decreases rapidly. This can be detected easily from the large discrepancy in the modulation depths $m_{2\omega }^{-}$ and $m_{2\omega }^{+}$.

It is worth noting that if the measurements of $m_{2\omega }^{-}$ and $m_{2\omega }^{+}$ were made for two orientations of the polarizer α = −45° and +45°, with the analyzer azimuth unchanged, theoretically it should again lead to Formula (12). Unfortunately, in real systems, the rotation of the polarizer may slightly change the direction of the light beam passing through the sample in the cuvette and quarter-wave plate, which means that we cannot be sure that $\gamma -q\Gamma_{0}$ is kept constant.

## 3. Experiment

**Figure 2:**
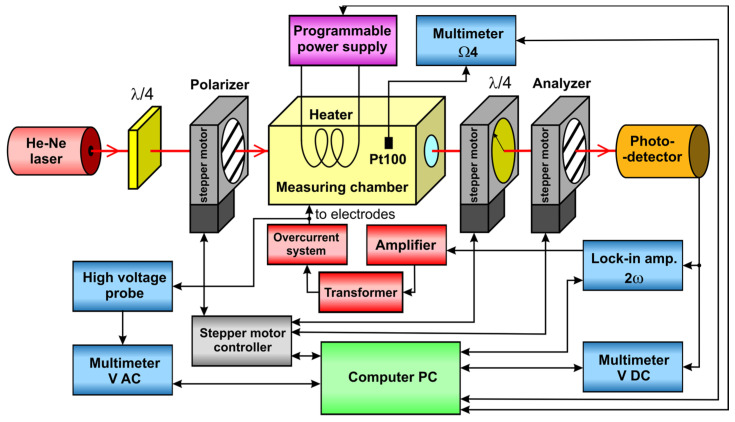
Block diagram of experimental setup for electro-optical measurements.

Figure 2 shows a diagram of our experimental setup for electro-optical measurements. The He-Ne laser used was Lasos LGK 7665 P with a wavelength of 632.8 nm and power of 15 mW. The first of the two quarter-wave plates (λ/4) was used to change the linear polarization of the emitted light to circular polarization. The polarizer, the second quarter-wave plate, and the analyzer were rotated independently using three Thorlabs NR/360 nanorotator stages connected to a Thorlabs BSC203 three-channel stepper motor controller. The oil sample was poured into a glass spectrophotometric cuvette with plane-parallel electrodes (L=49.16 mm) made from stainless steel with teflon spacers (d=2.82 mm). The cuvette was enclosed in a measuring chamber designed to control temperature.

The light passing through the analyzer was directed at a Thorlabs PDA100A-EC photodetector, which generated a voltage *U* proportional to the intensity *I* of the incident light. The constant component $U_{0}$ of the voltage *U* was measured using a Keithley 2000 multimeter. The voltage $U_{2\omega }$ and phase $\varphi_{2\omega }$ of the second harmonic component were measured using an EG&G 7265 lock-in amplifier. The lock-in amplifier was also used as the source of a modulating signal, which was amplified using a Yamaha A-S501 amplifier and a TELTO TSZ 90 VA high-voltage transformer. The high voltage $U_{m}$ applied to the electrodes was measured using a Keithley 2000 multimeter equipped with a Tektronix P6015A high-voltage probe.

The entire measurement procedure was controlled by a single PC using our own software. The procedure consisted of five loops, where each subsequent loop was nested within the previous loop:

1. In the outermost loop, the temperature of the sample was increased from 25 °C to 80 °C in 5 °C steps. After each temperature change, the control program waited 6 h for a stable temperature, with fluctuations smaller than ±0.01 °C.
2. The program set all eight combinations of two polarizer azimuths of −45° and +45°, two quarter-wave plate azimuths of 0° and 90°, and two analyzer azimuths of −45° and +45°.
3. The frequency of the modulating signal was set to successive values of 67, 167, 217, 317, 417, 517, 617, 717, 817, 917, and 1017 Hz. These frequencies were chosen to avoid all harmonics of the 50 Hz mains.
4. The output voltage of the oscillator in the lock-in amplifier was set to 15 selected levels, which corresponded to an increase in the voltage $U_{m}$ applied to the electrodes from approximately 800 to 2500 V RMS.
5. The readings of $U_{m}$, $U_{0}$, $U_{2\omega }$, and $\varphi_{2\omega }$ were repeated 15 times, and the results were averaged.

The procedure described above required approximately 6 days for each oil sample.

## 4. Results

**Figure 3:**
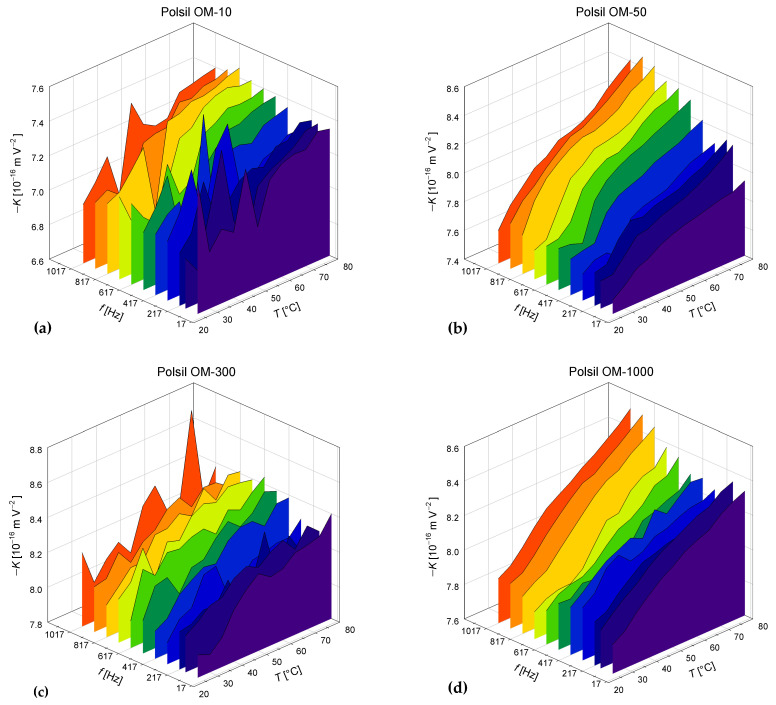

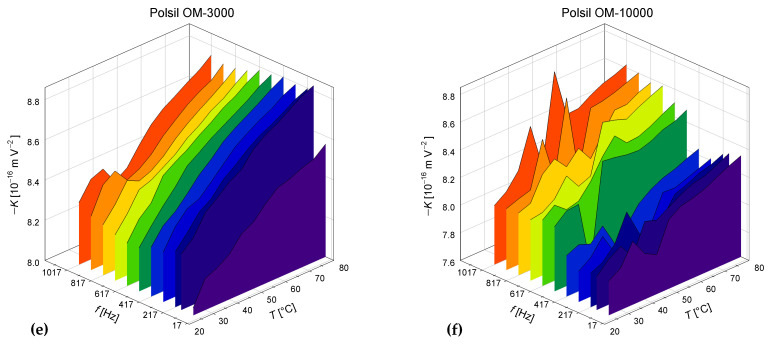
Temperature and frequency dependencies of the Kerr coefficient *K* in methyl silicone oils POLSIL^®^ OM with viscosities of (**a**) 10 cSt; (**b**) 50 cSt; (**c**) 300 cSt; (**d**) 1000 cSt; (**e**) 3000 cSt; and (**f**) 10,000 cSt.

The temperature and frequency dependencies of the Kerr coefficient measured for the six methyl silicone oils with various viscosities are shown in Figure 3. All obtained values are negative. Although the dependencies have some local disturbances, there is a clear positive correlation between the absolute value of the Kerr coefficient and temperature visible for all samples. Because such a dependence is unusual and the range of observed changes is relatively narrow, we made every effort to exclude the influence of the measuring equipment or procedure. In particular, we performed measurements for other liquids and solids, which confirmed the typical decreasing dependence of *K* on *T*. Moreover, all electro-optical measurements were repeated for eight optimal configurations of the measurement system, including two polarizer azimuths of −90° and +90°, two quarter-wave plate azimuths of 0° and 90°, and two analyzer azimuths of −90° and +90°. The results obtained for configurations differing only in the azimuth of the analyzer were averaged using the geometric mean (12), which, according to the model described in Section 2, should make the results independent of the deviations of the total phase shift in the measurement system from the ideal value of 90°. The four geometric means obtained had very similar values, and their arithmetic means are presented in this paper.

The electro-optical properties of substances are traditionally described by several coefficients whose temperature dependencies may vary significantly. In addition to the Kerr coefficient, electro-optic coefficients are commonly used, which are defined by expanding the components $B_{ij}$ of the impermeability tensor at optical frequencies into the following power series [23]:

(13) $$B_{ij}=\delta_{ij}n_{i}^{-2}+r_{ijk}E_{k}+g_{ijkl}E_{k}E_{l}+\cdots \;,$$

where $\delta_{ij}$ is the Kronecker delta; $n_{i}$ represents the field-free principal refractive indices; $r_{ijk}$ and $g_{ijkl}$ are the coefficients of the linear and quadratic electro-optic effects, respectively; and $E_{k}$ represents the components of the applied low-frequency electric field.

Assuming the form of the $\left[g_{ijkl}\right]$ tensor for an isotropic medium with ∞∞ symmetry [23] and the measurement configuration given in Section 2, we obtain the relation

(14) $$g_{1111}-g_{1122}=-\frac{2\lambda }{n^{3}}K,$$

where λ is the wavelength of light. It should be noted that when the orientational ordering of the molecules in the applied field is slow compared to the period of the field oscillation, this can be considered as lowering the symmetry to ∞2. In this case, by neglecting small differences between the $n_{i}$ coefficients, we should input the effective electro-optic coefficient $q_{1111}-q_{3311}$ instead of $q_{1111}-q_{1122}$ in Equation (14).

The quadratic electro-optic effect can also be described by the so-called intrinsic coefficients defined in terms of electric polarization instead of the applied field [12,13], as follows:

(15) $$f_{1111}-f_{1122}=\frac{g_{1111}-g_{1122}}{\varepsilon_{0}^{2}{\left(\varepsilon -1\right)}^{2}},$$

where $\varepsilon_{0}$ is the vacuum permittivity and ε is the low-frequency dielectric constant.

In addition, we consider the molecular Kerr constant $K_{m}$, which is related to the usual Kerr coefficient *K* in the following formula [1]:

(16) $$K_{m}=\frac{6n\;V_{m}\lambda }{{\left(n^{2}+2\right)}^{2}{\left(\varepsilon +2\right)}^{2}}K,$$

where $V_{m}$ is the molar volume.

To investigate the temperature dependence of the $g_{1111}-g_{1122}$, $f_{1111}-f_{1122}$, and $K_{m}$ coefficients, the temperature dependence of the refractive index n(T) must be known. As the data available in the literature are only fragmentary, we performed our measurements using an Abbe refractometer. We found that the refractive index decreased linearly with increasing temperature for all samples of methyl silicone oils measured over a temperature range of approximately 20–80 °C (Table 2).

To calculate the $f_{1111}-f_{1122}$ and $K_{m}$ coefficients, the values of ε as a function of temperature and frequency were also required. Because data are available only for selected temperatures and frequencies, we performed our own measurements using the LCR meter GW INSTEK LCR-6100. We found that the dielectric constant of all the oil samples showed no frequency dependence in the tested range from 67 Hz to 1017 Hz. The observed temperature dependencies clearly decreased for all samples. Because these dependencies could not be described satisfactorily by a linear function or a Curie–Weiss function, we used second-degree polynomials (Table 3).

The molecular Kerr constant $K_{m}$ depends on the molar volume $V_{m}$. Because of the lack of data in the literature, we calculated $V_{m}(T)=M/\rho (T)$ using the temperature dependence of the density of methyl silicone oils $\rho (T\;[{}^{\circ}C])=\rho (25\;{}^{\circ}C)/[1+0.00092\;(T-25)+0.00000045\;{\left(T-25\right)}^{2}]$ given in [17] and the densities of oils at 25 °C [24]. The atomic mass *M* was calculated based on the chemical structure shown in Figure 1 and the average number *m* of dimethylsiloxane segments. Because the manufacturer does not provide such data for oils with viscosities of 300 cSt and 3000 cSt, we estimated *m* using the smoothed dependence of *m* on the viscosity fitted to the data for seven other oils (Table 1).

**Figure 4:**
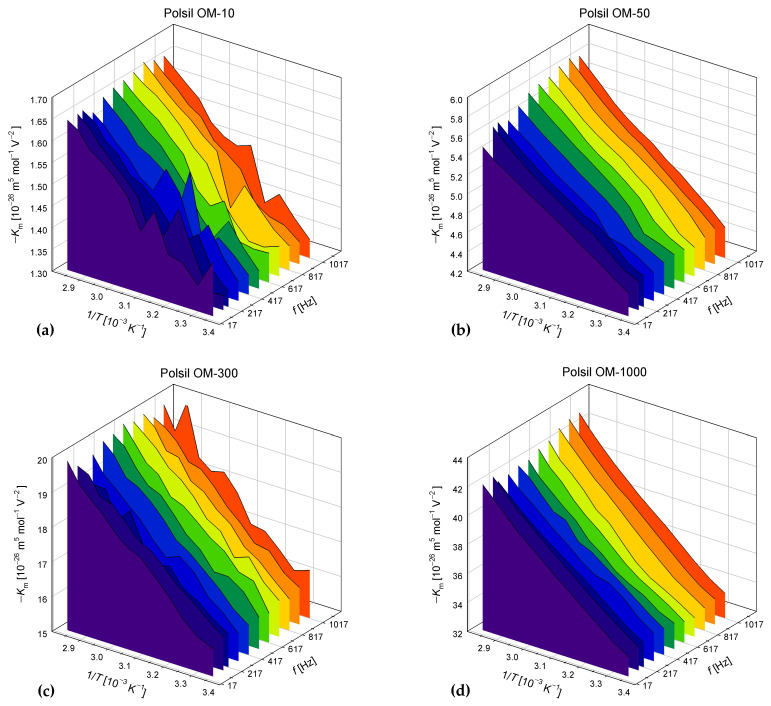

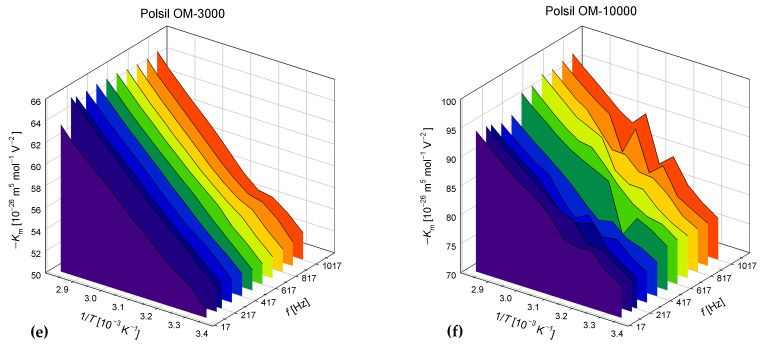
Temperature and frequency dependencies of the molecular Kerr constant *K*_m_ in methyl silicone oils POLSIL^®^ OM with viscosities of (**a**) 10 cSt; (**b**) 50 cSt; (**c**) 300 cSt; (**d**) 1000 cSt; (**e**) 3000 cSt; and (**f**) 10,000 cSt.

The obtained the dependencies of $K_{m}$ on the frequency and reciprocal of temperature, which are presented in Figure 4. The temperature dependencies can be approximated by the function $K_{m}=A_{0}+A_{1}/T$ predicted for non-polar liquids. However, it is surprising that $A_{0}<0$ and $A_{1}>0$ for all oil samples, which cannot be correctly described by Buckingham’s classical theory (see Section 5—Discussion). The coefficient $K_{m}$ depends strongly on the oil viscosity, which results from different values of the molar volume in Formula (16), while the values of $K_{m}/V_{m}$ turn out to be almost independent of the viscosity. The values of the $A_{1}/A_{0}$ ratio are within a narrow range from –192 to –162 K for all oil samples and frequencies.

**Figure 5:**
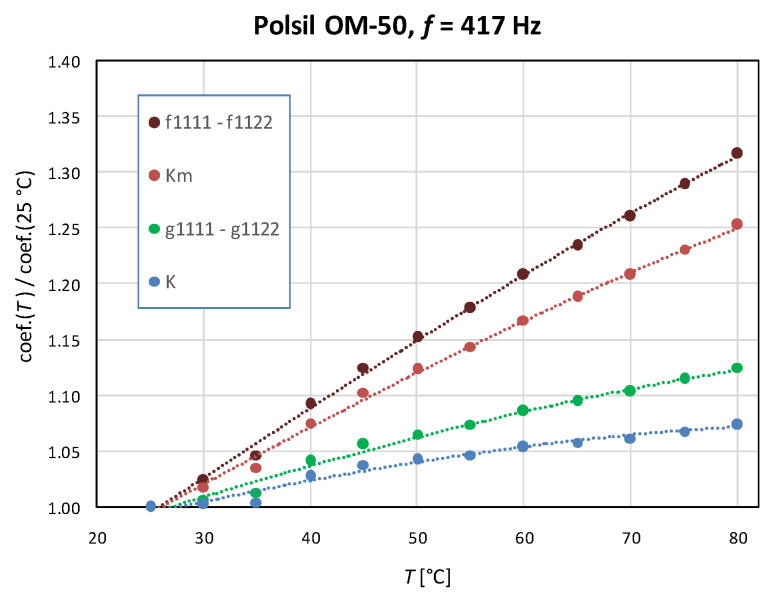
Example comparison of temperature dependencies of the Kerr coefficient *K*, quadratic electro-optic coefficient defined in terms of an applied field $g_{1111}-g_{1122}$ and in terms of polarization $f_{1111}-f_{1122}$, and the molecular Kerr constant $K_{m}$ for methyl silicone oil with a viscosity of 50 cSt in a 417 Hz modulating field.

Owing to the decreasing temperature dependence of the refractive index for all oils, the coefficient $g_{1111}-g_{1122}$ increases noticeably faster with the increasing temperature than *K* (Figure 5). Moreover, the decreasing temperature dependence of the dielectric constant results in an even stronger increasing temperature dependence of the intrinsic electro-optic coefficient $f_{1111}-f_{1122}$ than that obtained for $g_{1111}-g_{1122}$. The temperature dependence of $K_{m}$ is always intermediate between those observed for $g_{1111}-g_{1122}$ and $f_{1111}-f_{1122}$.

Due to the large amount of data and similar dependencies obtained for all individual samples and frequencies, only some results are included here. All obtained values of the coefficients K, $g_{1111}-g_{1122}$, $f_{1111}-f_{1122}$, $K_{m}$, $A_{0}$, and $A_{1}$ are available in Tables S1–S5, respectively, located in the Supplementary Materials.

## 5. Discussion

Despite the huge differences in the viscosity of the methyl silicone oil samples, all values for the Kerr coefficient obtained at λ = 632.8 nm fall within a relatively narrow range from −8.83 × 10^−16^ to −6.79 × 10^−16^ m V^−2^ across all studied temperatures and frequencies. To our knowledge, these values are lower than almost all previously reported ones, including remarkably low values such as 12.3 × 10^−16^ m V^−2^ for methyl n-Propyl Ketone, 13.6 × 10^−16^ m V^−2^ for butyraldehyde [3], or 18 × 10^−16^ m V^−2^ for fresh transformer oil at room temperature [8]. A similar value of 7.63 × 10^−16^ m V^−2^ at room temperature was reported only for 1,4-dioxane [25], which is not suitable for being used as an immersion liquid due to its hygroscopicity, volatility, and toxicity. Our values for the Kerr constant are also at least two orders of magnitude smaller than those typical for the solid crystals used in nonlinear optics, for example, *K* = 8.3 × 10^−14^ m V^−2^ for the KDP crystal at room temperature and λ = 632.8 nm (calculated according to Formula (14) for $g_{1111}-g_{1122}$ given in [16]). Therefore, we expect that the electro-optical measurements of solid crystals immersed in methyl silicone oil will not be significantly affected by the fringing electric field around the electrodes on the crystal.

The experimental temperature dependencies of $K_{m}$ shown in Figure 4 match the theoretical dependence (1) with $A_{2}=0$ predicted for non-polar molecules. However, the interpretation of negative $A_{0}$ in combination with positive $A_{1}$ values resulting from our measurements may raise considerable doubts. Although negative values of the Kerr constant in liquids are already known from the literature (e.g., [25,26,27]), the increasing temperature dependence of the absolute value of $K_{m}$ has probably not been reported. According to Buckingham’s theory for non-polar substances [1],

(17) $$K_{m}=\frac{2\pi N}{405}\left[2\gamma_{\alpha \alpha \beta \beta }^{\left(1\right)}+\frac{3}{kT}\left(\sum_{s=1}^{3}\alpha_{s}\alpha_{s}^{\left(0\right)}-3\alpha \alpha^{\left(0\right)}\right)\right],$$

where $\alpha_{s}$ and $\alpha_{s}^{\left(0\right)}$ are the principal polarizabilities of a molecule in the optical frequency and static/low-frequency fields, respectively; $\alpha =\left(\alpha_{1}+\alpha_{2}+\alpha_{3}\right)/3$, $\gamma_{\alpha \alpha \beta \beta }^{\left(1\right)}$ are the components of the hyperpolarizability tensor; *N* is Avogadro’s number; and *k* is the Boltzmann constant. Fitting the dependence (17) to our experimental data leads to relatively large negative values of the temperature-independent term and smaller positive values of the term proportional to 1/*T*. This result seems questionable and is contrary to the data presented, for example, in [1], which show that the temperature-independent term should be much less important at room temperature. The theoretical dependence (17) was derived under the assumption that long-range ordering does not contribute to $K_{m}$ because, for an assembly of non-polar molecules, the interaction potential energies do not depend on their orientation. Another assumption is to use the $\alpha_{s}$ and $\alpha_{s}^{\left(0\right)}$ polarizabilities as temperature-independent molecular constants. However, both assumptions seem inconsistent with the recent observations of efficient hydrogen bonding in polydimethylsiloxanes, where local ordering of chains occurs with more or less parallel alignment of individual chain backbones in lamellar domains [28]. Because hydrogen bonds are relatively weak, long-range ordering disappears with increasing thermal motions.

The intrinsic electro-optic coefficients $f_{ijkl}$, i.e., the coefficients defined in terms of induced electric polarization, are widely used to describe solid crystals because they show less material dependence [12,13] and less temperature dependence [14,16,29] than the $g_{ijkl}$ coefficients. The intrinsic coefficients have probably not been applied to liquids. The results obtained in this study show that the conclusions formulated earlier for solid crystals cannot be simply extended to methyl silicone oils, in which the temperature dependence of the $f_{1111}-f_{1122}$ coefficient is stronger than that of the *K*, $K_{m}$, and $g_{1111}-g_{1122}$ coefficients. It is also worth noting the significant difference between the temperature dependence of the *K* and $K_{m}$ coefficients. Because the values of $K_{m}$ are more difficult to find, dependence (1) has been applied directly to *K* in some previous studies, and it has no theoretical justification.

The maximum measurement uncertainty, estimated based on the accuracy of the measuring instruments used, does not exceed 1% for the *K* and $g_{1111}-g_{1122}$ coefficients or 1.5% for $f_{1111}-f_{1122}$. However, we observed that the effect of the frequency of the modulating field clearly exceeds these uncertainties. The frequency effect manifests as slightly lower absolute values of the Kerr coefficient for the lowest frequencies (Figure 3), while at higher frequencies, we only observe fluctuations specific to a given oil sample. These fluctuations are repeatable in all series of measurements made for different orientations of the polarizer and quadrant of the plate. Because no theoretical general dependence of the Kerr coefficient on frequency is currently known for liquids and there is insufficient experimental data for other liquids, we cannot formulate any general rule.

We observed slightly smaller values of the Kerr constant for oils with the lowest viscosities, especially for the Polsil OM-10 oil, which confirms the previous results presented in [10]. This effect is probably due to the lower density of the oils with the lowest viscosities and atomic masses, while the density becomes almost independent of the atomic mass for viscosities above 300 cSt [24]. However, it should be noted that these are average data, and, in practice, oil of a given viscosity can be produced by mixing oils of other viscosities, which may slightly affect its density.

## 6. Conclusions

We performed electro-optical measurements of methyl silicone oils with viscosities from 10 to 10,000 cSt (at 25 °C) at temperatures from 25 to 80 °C and frequencies from 67 to 1017 Hz. All obtained values of the Kerr coefficient were negative and fell within a relatively narrow range from −8.83 × 10^−16^ to −6.79 × 10^−16^ m V^−2^. The coefficient $g_{1111}-g_{1122}$ of the quadratic electro-optic effect ranged from 3.16 × 10^−22^ to 4.23 × 10^−22^ m^2^ V^−2^, which are among the lowest values reported for liquids thus far. Methyl silicone oils also offer several other advantageous properties, such as a low dielectric constant, high electrical resistivity, transparency, chemical stability, non-volatility, non-flammability, hydrophobicity, and non-toxicity, making them very suitable as immersion liquids for electro-optical studies of solid crystals and other solid materials.

Unusually, increasing temperature dependencies were observed for the absolute values of the Kerr coefficient, molecular Kerr constant, and quadratic electro-optic coefficient of all oil samples over the entire studied temperature range. Such dependencies have not been reported for other liquids and cannot be properly described by Buckingham’s classical molecular theory of the Kerr effect. Moreover, the intrinsic quadratic electro-optic coefficient (i.e., the coefficient defined in terms of induced polarization) increased even faster with increasing temperature than any other electro-optical quantities. This result completely contrasts the results of previous studies, in which particularly weak temperature dependencies of various intrinsic coefficients were observed for many solids.

To investigate the influence of several factors, we needed a method that could ensure both rapid and precise electro-optical measurements at the same time. The improved dynamic polarimetric method proposed in this paper used two operating points on the transmission characteristics of the electro-optical modulator, and only their relative positions needed to be controlled carefully. Because the change in the position of the operating point can be controlled precisely, easily, and quickly using a rotary stepper motor, our approach is more effective than traditional dynamic methods, in which measurement accuracy strongly depends on control over the absolute position of a single operating point.

## Figures and Tables

**Table 1:** Approximate relationship between the viscosity of a methyl silicone oil, its molecular weight, and the average number *m* of dimethylsiloxane segments [17].

Viscosity at 25 °C [cSt]	Molecular Weight	*m*
10	1200	15
50	3800	50
100	6000	80
500	17,000	230
1000	28,000	375
5000	49,000	650
10,000	62,000	840

**Table 2:** Parameters of the linear dependence n(T [°C])=n(0)+aT fitted by the least squares method to the experimental values of the refractive index *n* for methyl silicone oils of various viscosities. To compare the values calculated from the linear function with experimental values, we used the mean absolute error $MAE=\sum_{i=1}^{N}\left|n_{exp}-n_{calc}\right|/N$.

Viscosity at 25 °C [cSt]	*n*(0)	*a* [10^−4^ °C^−1^]	*MAE*
10	1.4092	−4.24	0.0002
50	1.4129	−4.00	0.0003
300	1.4133	−3.65	0.0003
1000	1.4142	−3.96	0.0002
3000	1.4142	−3.96	0.0001
10,000	1.4139	−3.90	0.0003

**Table 3:** Parameters of the quadratic dependence $\varepsilon (T\;[{}^{\circ}C])=AT^{2}+BT+C$ fitted by the least squares method to the experimental values of the dielectric constant ε for methyl silicone oils of various viscosities. Mean absolute error $MAE=\sum_{i=1}^{N}\left|\varepsilon_{exp}-\varepsilon_{calc}\right|/N$.

Viscosity at 25 °C [cSt]	*A* [10^−5^ °C^−2^]	*B* [°C^−1^]	*C*	*MAE*
10	1.054	−0.00444	2.7137	0.0016
50	1.486	−0.00527	2.8289	0.0011
300	0.259	−0.00475	2.8511	0.0022
1000	−0.083	−0.00403	2.8531	0.0011
3000	0.933	−0.00499	2.8613	0.0008
10,000	0.507	−0.00465	2.8651	0.0014

## Data Availability

The data presented in this study are available in the Supplementary Materials.

## Supplementary Materials

The following supporting information can be downloaded at: https://www.mdpi.com/article/10.3390/ma17081850/s1, Table S1: Experimental values of the Kerr coefficient *K* in methyl silicone oils POLSIL^®^ OM; Table S2: Experimental values of the quadratic electro-optic coefficient *g*_1111_ − *g*_1122_ defined in terms of an applied field in methyl silicone oils POLSIL^®^ OM; Table S3: Experimental values of the intrinsic quadratic electro-optic coefficient *f*_1111_ − *f*_1122_ defined in terms of electric polarization induced in methyl silicone oils POLSIL^®^ OM; Table S4: Experimental values of the molecular Kerr constant *K*_m_ in methyl silicone oils POLSIL^®^ OM; Table S5: Values of the *A*_0_ and *A*_1_ coefficients in the approximation of the temperature dependence of the molecular Kerr constant *K*_m_ = *A*_0_ + *A*_1_/*T* for methylsilion oils Polsil^®^ OM.
